# Short-Term Effects of a Conditioning Telerehabilitation Program in Confined Patients Affected by COVID-19 in the Acute Phase. A Pilot Randomized Controlled Trial

**DOI:** 10.3390/medicina57070684

**Published:** 2021-07-03

**Authors:** Cleofas Rodriguez-Blanco, Juan Jose Gonzalez-Gerez, Carlos Bernal-Utrera, Ernesto Anarte-Lazo, Manuel Perez-Ale, Manuel Saavedra-Hernandez

**Affiliations:** 1 Department of Physiotherapy, Faculty of Nursing, Physiotherapy and Podiatry, University of Seville, 41009 Seville, Spain; cleofas@us.es; 2 Department of Nursing, Physiotherapy and Medicine, University of Almeria, 04120 Almeria, Spain; clinicafisiosur@gmail.com (J.J.G.-G.); clinicasaavedra@yahoo.es (M.S.-H.); 3 Doctoral Program in Health Sciences, University of Seville, 41009 Seville, Spain; anartelazo.ernesto@gmail.com; 4 Physician in Intensive Medicine in the Spanish Army, Health Support in the Naval Base of Rota, 11520 Cádiz, Spain; manuel.manuelale@gmail.com

**Keywords:** COVID-19, physical therapy, telerehabilitation, exercise therapy

## Abstract

*Background and objectives*: The COVID-19 pandemic has become a challenge for health systems and, specifically, to physical therapists obligated to adapt their job and stop face-to-face consultations. In this situation, therapeutic exercise has been implemented in different COVID-19 patients. This study evaluated the feasibility and effectiveness of a novel therapeutic exercise program through telerehabilitation tools in COVID-19 patients with mild to moderate symptomatology in the acute stage. *Materials and Methods*: A total of 40 subjects were randomized an experimental group, based on muscle conditioning, and in a control group, who did not perform physical activity. Thirty-six subjects, 18 in each group, completed the one-week intervention. We measured the six-minute walking test, multidimensional dyspnoea-12, thirty seconds sit-to-stand test, and Borg Scale. *Results*: Both groups were comparable at baseline. Statistically significant improvement between groups (*p* < 0.05) in favor of the experimental group was obtained. No differences between gender were found (*p* > 0.05). Ninety percent adherence was found in our program. *Conclusion*: A one-week telerehabilitation program based on muscle toning exercise is effective, safe, and feasible in COVID-19 patients with mild to moderate symptomatology in the acute stage.

## 1. Introduction

In December 2019, a highly contagious and rapidly spreading respiratory illness was introduced into all countries. Finally, a pandemic due to SARS-CoV-2, causing the disease COVID-19, was declared in March 2020 [1]. To avoid the virus spreading, home confinement proposed by the World Health Organization (WHO) as the best option for those infected [2].

The clinical spectrum of COVID-19 varies from asymptomatic forms to clinical conditions that require mechanical ventilation and admission to intensive care unit due to respiratory failure [3]. Among those subjects with mild conditions, the main symptoms are fever, cough, dyspnea, headache, sore throat, and rhinorrhea [4]. It has already been argued that the host’s immune strength plays a key role against COVID-19 [5] as a dysregulated immune response has been related to medium-term effects of SARS-CoV-2 on multiple organs, exercise capacity, and quality of life [6].

In that sense, it has been argued that continuing physical activity during confinement to stay healthy and maintain immune system function is a reasonable health rationale [7], as confinement generates serious problems in the physical and nutritional state of patients [8]. Indeed, exercise can play an essential role against respiratory infections and disorders, such as pneumonia and acute respiratory distress syndrome (ARDS), which are two common disorders with COVID-19 [9,10] and, in people in the lockdown period, it is strongly recommended to decrease the risk factors of COVID-19, improving immune and respiratory systems’ function to allow better body response against the disease [11].

However, traditional rehabilitation has involved human interaction and physical contact, leading to increased transmission and contagiousness. To face this problem, telerehabilitation has increased in importance. Indeed, it has been demonstrated that a session of exercise through a telerehabilitation system is feasible [12], and it has been proposed as a way of managing COVID-19 [13].

Therefore, we conducted a pilot randomized controlled trial to analyze the feasibility and safety of this intervention in the acute phase and evaluate the financial, technical, administrative, or logistic feasibility of a full-scale study, including issues of data collection, protocol adherence, and obtaining values to calculate a sample size of the full-scale study.

## 2. Materials and Methods

### 2.1. Trial Design

The pilot trial design was a randomized, controlled, parallel, double-blind, two-arm clinical trial of treatment. Trial registration: Brazilian Clinical Trial Registry, RBR-4j9wkbz. It was registered on 5 April 2021.

### 2.2. Sample Selection

Patients were recruited through an informative text message transmitted on social networks (WhatsApp, Facebook, Instagram, Twitter, LinkedIn), TV channels such as Deutsche Welle, radio programs, and newspapers. They were contacted by a general message that informed the possibility of participating in a physiotherapy study; all those interested were reported later in greater detail. The recruitment was in Spain. Therefore, any patient resident in Spain could participate in this research if he/she was a positive for SARS-CoV-2 through PCR tests (polymerase chain reaction) and antigen-test, classified by each region’s epidemiology services. To start the study, the patient had to sign informed consent on the webite www.fisiosurid.com/covid-19/ (accessed on 19 October 2020). Later, they were selected according to the listed eligibility criteria. The study took place at patients’ homes; evaluators carried out all measurements on the first, seventh, and fourteenth days. All patients were instructed and telematically controlled by the study evaluator, who provided patients the necessary assessment materials described below (the “Outcome Measurements” section).

We excluded those patients who required derivation of hospital care. The criteria were based on those published by the Spanish Society of Family and Community Medicine (SEMFYC) and can be checked in the “Exclusion Criteria” section [13].

### 2.3. Inclusion Criteria

The following were the inclusion criteria:

1. Age 18–75 years.
2. Patients with positive polymerase chain reaction (PCR) test and/or antigen-test for SARS-CoV-2 in the last forty days were in home confinement.

### 2.4. Exclusion Criteria

The following were the exclusion criteria:

Patients with chronic lung conditions, chronic kidney disease, and chronic neurological disorders. Patients were suffering from hypertension and cardiovascular conditions without medical treatment. Patients affected with grade III osteoporosis, with the acute phase of rheumatologic diseases, and with the acute phase of disc abnormalities.

Patients who had respiratory conditions in the last 12 months, recent musculoskeletal disorders, were not fully recovered from their injuries, received physical therapy treatment in the previous three months, and were affected with chronic mental and/or psychological disturbances.

Red flags of severe conditions (night pain, painful muscle spasm, loss of involuntary weight, and symptom mismatch).

Patients classified as moderate/severe cases based on the Spanish Society of Family and Community Medicine (SEMFYC) [14].

### 2.5. Interventions

Patients performed exclusively assigned therapeutic exercises or sedentary activities (depending on randomized allocation to the study groups) and could not combine with other physical therapy or sports physical activity. Any interference in the treatment was grounds for exclusion. Participants were asked in the daily contact sessions if they had carried out any action that can be considered in the treatment. If participants were required to combine the intervention with medications, it was registered. Exercise monitoring was developed through telerehabilitation tools, emerging technology through which medical rehabilitation care can be provided from a distance. Patients were encouraged to ultimately carry out treatment and follow-up through videoconferences that enabled them to improve their health status through their effort and reduced the rate of loss to follow-ups and dropouts.

#### 2.5.1. Group 1: Non-Specific Conditioning Exercise Program (NTEP)

The NTEP consisted of 10 exercises based on non-specific toning exercises of resistance and strength to try to improve the physical deconditioning and physiological deterioration that implies. Moreover, it has been demonstrated that exercise therapy produces many benefits in the immune/defense system. The exercises are available at https://www.fisiosurid.com/ejercicios-proyecto-covid/ (accessed on 19 October 2020). It was carried out once a day, for seven days, at the patient’s home. Depending on the score obtained on the Borg scale (BS) during the assessment, patients performed 4 (BS 7_10), 8 (BS 5_7), or 12 (BS 1_5) repetitions per exercises and day; these repetitions took 10, 20, and 30 min, respectively. The exercise program was reinforced by a physical therapist at least two times (if the patient does not require further attention) through telematic control by videoconference with each patient. Additionally, patients received a text message daily, asking about the exercises and as a follow-up method and improving adherence. It has been demonstrated that exercise therapy produces many benefits in the immune/defense system. Owing to the established relationship between this system and COVID-19 effects, we decided to include a group of unspecific exercises and analyze how patients benefited from this exercise [15,16]. We also try to avoid physical deconditioning, with the physiological deterioration it implies [17,18].

#### 2.5.2. Control Group (CG)

The patients in this CG underwent the two assessments on days 1 and 7. These assessments were carried out by a physiotherapist who was unaware of the group to which the patient belonged. Once the data from the different evaluations had been obtained, the patients were taught group 1.

### 2.6. Outcome Measures

The data were collected by personnel attached to the research group who had previously been instructed in the procedures to follow and did not know the group to which the patients belonged; the information sent by the patients was stored and classified; the evaluators transferred the numerical values to an Excel table. The Excel tables are encrypted, and only the evaluators and the leading researchers had access to it. This information was updated through a secure cloud. All outcomes were measured using WhatsApp or by email on the first and the seventh day.

Six-minute walk test (6MWT) [19]. The patient’s smartphone recorded the number of steps through the app “StepsApp”. This test can determine the functional state correctly.

Thirty seconds sit-to-stand test (30STST) [20,21]. This test has been demonstrated to be a valid and reliable tool to assess peripheral muscle performance of lower limbs.

Borg scale (BS). [22]. The Borg scale of perceived effort measures the entire range of activities that the individual perceives when exercising. This scale gives criteria to make adjustments to the intensity of exercise, that is, to the workload, thus forecasting and dictating the different exercise powers in sports and medical rehabilitation. Patients completed the BS at the end of the 30STST.

### 2.7. Randomization

Patients were divided into two groups using balanced randomization, carried out with free software (http://www.randomized.org/) (accessed on 12 October 2020). The principal investigator and auditor only performed the randomization sequence. No participant in the study had access to the randomization sequence, which was hidden and saved, to guarantee correct randomization with security.

### 2.8. Blinding

Evaluators and patients in the study were blinded during the entire process. The evaluator was unaware of the study objectives and the randomized distribution of patients to study groups, and he did not have access to the randomization sequence. Meanwhile, although blinding for patients ultimately could not be achieved, subjects were unaware of other treatment modalities. They did not know if they belonged to the intervention or sham group.

### 2.9. Statistical Analysis

We carried out the statistical analysis through a descriptive analysis of the data before the intervention, as a baseline, applying the Shapiro–Wilk normality test for the quantitative variables (Table 1 and Table 2). The within-group analysis was performed based on Shapiro–Wilk *p*-values previously obtained by using the paired *t*-test or Wilcoxon Z-test (Table 3). The between-group analysis was performed based on Shapiro–Wilk *p*-values previously obtained by applying the independent t-test or Mann–Whitney U-test (Table 3, Table 4). The statistical analysis was conducted at a 95% confidence level. A *p*-value of less than 0.05 was considered statistically significant in all analyses. Statistical analysis was carried out using SPSS v.26.0 (IBM, Armonk, NY, USA).

## 3. Results

**Figure 1:**
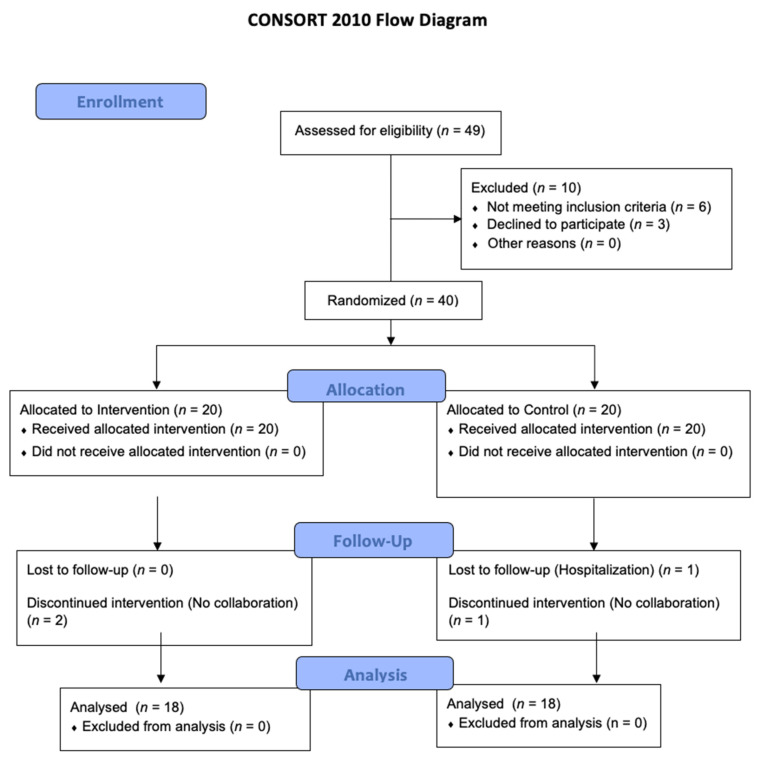
CONSORT flow diagram.

After conducting 49 evaluations, 40 patients started the pilot study, while 36 patients (90%), 19 women and 17 men, completed the 7-day intervention (pilot study period, 6–13 April 2021) and were included in the analysis. The flow diagram can be seen in Figure 1 (see Supplementary Materials).

Both groups were comparable at baseline, the exercise group (*n* = 18) and the control group (*n* = 18). Table 1 and Table 2 summarize pre- and post-intervention data for the groups.

The 7-day intervention of exercises resulted in a statistically significant improvement between groups (*p* < 0,05) (Table 3). No differences between gender were found (*p* > 0,05) (Table 4).

Regarding the EG, we observed within-group differences in all of the variables studied (Table 3), although we have seen the most remarkable differences in the BS variable. We have not observed intragroup differences in the variables of the CG (*p* > 0.05) in BS and 6MWT, but there are differences in 30STST. The intergroup analysis shows us significant differences between the EG versus the CG (Table 3) in all variables, mainly in the BS variable (*p* < 0.001).

## 4. Discussion

The COVID-19 pandemic has become a challenge for health systems and, specifically, to physical therapists obligated to adapt their job and stop face-to-face consultations. In addition, physical function, exercise, and immune system have been argued to be related to each other as well as to COVID-19 management [23,24]. In this randomized pilot trial, we have demonstrated that it is possible to recruit mild to moderate COVID-19 patients through social media to be included in a telerehabilitation exercise program based on muscle toning.

Different tests have been suggested as possible ways to measure physical function in patients with COVID-19 at home [25]. In our study, we assessed 6MWT, 30STST, and BS, and our results showed that all these variables significantly changed, reducing the level of perceived exertion, improving the capacity of these patients in basic physical activities (*p* < 0.05) such as walking or sitting and getting up from a chair (*p* < 0.001).

Compared with controls, exercise intervention improved all outcomes so that patients with COVID-19 were significantly enhanced. Hence, it can be suggested that different mechanisms may play a role in rehabilitation and improvement of symptomatology and function in these patients. The BS decreases significantly in EG when compared with CG (*p* < 0.001).

The data obtained in the 6MWT show us that there are significant differences (*p* = 0.026) between groups. Therefore, the 6MWT evidence physical benefits in relation to cardiopulmonary rehabilitation. Regarding the 30STST, we observed significant differences in both groups, exercise (*p* = 0.011) and control (*p* = 0.026). We must point out that the statistical significance in the control group is against clinical improvement. The sedentary group one week after starting the study fails to improve and reduces its level of strength, with substantial differences with respect to the exercise group.

Regarding the analysis by gender, we did not obtain notable differences. There is a trend towards significance in the 30STST variable in favor of men, indicating that they could recover the strength parameters earlier; however, this finding is not conclusive.

Different mechanisms could explain why therapeutic exercise may be favorable for these patients, such as improvements in glucose metabolism and mitochondrial function [26,27,28], strengthening of the immune system [23,24], and improvements in respiratory function [11]. Therefore, rehabilitation has been recognized as a treatment option in patients with mild and moderate clinical symptoms of COVID-19 [29].

This pilot study also aimed to assess the feasibility and safety of patients with COVID-19. From that perspective, we observed encouraging results. Only three subjects who started the study did not complete the intervention and evaluation, with a loss rate of 10%. Two of these losses were due to a lack of collaboration of subjects, and the other one was due to hospitalization. Interestingly, this one was allocated in the control group. Although the sample size prevented conclusive findings, we proved that exercise intervention is safe and 90% of subjects adhered to the program.

There are several limitations to the study. First, given that this is a pilot study, it was most likely underpowered to detect an effect in physical function in patients with COVID-19. Second, we studied acute effects, and thus long-term effects cannot be evaluated based on the results in the study. Finally, clinical presentations differ between subjects, and although our intervention was implemented according to perceived effort, individualized treatment could not be provided.

However, we found that our intervention is feasible, safe, and consistent with our aims, so a more extensive study and future research are required to achieve definitive conclusions.

## 5. Conclusions

Therapeutic exercise implemented through telerehabilitation appears to provide a promising strategy for improving outcomes related to physical condition among patients with mild to moderate COVID-19 in the acute stage, indicating clinical benefits, adherence, and safety of the program. Our findings suggest not only clinical benefits, but also adherence and safety of the program.

## Figures and Tables

**Table 1:** Descriptives preintervention data. Data expressed as mean (standard deviation). BS: Borg scale; 6MWT: six-minute walking test; 30STST: 30-s sit to stand test; _1: Preintervention Data; _2: Postintervention Data; DIF_: Pre-post-Differences Data. The *p*-values come from the Shapiro–Wilk test.

	EXERCISE	CONTROL	
	(*n* = 18)	(*n* = 18)	
SEX			
WOMEN	9 (25)	10 (27.77)	
MEN	9 (25)	8 (22.22)	
AGE	39.39 (1174)	41.33 (12.13)	0.07
BS_1	4.78 (1.70)	4.78 (1.80)	0.122
BS_2	2.56 (0.85)	4.83 (1.54)	0.031
6MWT_1	440.17 (164.36)	379.78 (128.72)	0.616
6MWT_2	519.94 (135.33)	379.72 (136.01)	0.112
30STST_1	12.33 (4.81)	10.50 (2.25)	<0.001
30STST_2	13.83 (5.70)	9.94 (1.98)	<0.001
DIF_BS	−2.22 (1.30)	0.05 (1.25)	0.074
DIF_6MWT	79.77 (126.46)	−0.05 (26.38)	<0.001
DIF_30STST	1.50 (2.20)	−0.55 (0.92)	<0.001

**Table 2:** Descriptive data based on gender. M: mean; SD: standard deviation. BS: Borg scale; 6MWT: six-minute walking test; 30STST: 30-s sit to stand test; _1: Preintervention Data; _2: Postintervention Data; The *p*-values come from the Shapiro–Wilk test.

	GROUP								
	EXERCISE				CONTROL				
	WOMEN		MEN		WOMEN		MEN		
	M	SD	M	SD	M	SD	M	SD	*p*-Value
AGE	42.33	11.48	36.44	11.91	41.10	11.43	41.63	13.77	>0.05
BS_1	4.78	1.99	4.78	1.48	4.40	1.51	5.25	2.12	
BS_2	2.22	0.83	2.89	0.78	4.60	1.07	5.13	2.03	
6MWT_1	414.56	184.86	465.78	147.51	347.40	100.49	420.25	154.56	
6MWT_2	492.00	183.44	547.89	59.27	338.70	99.51	431.00	163.66	
30STST_1	12.78	6.63	11.89	2.20	10.80	1.99	10.13	2.64	
30STST_2	15.22	7.51	12.44	2.88	10.20	1.75	9.63	2.33	

**Table 3:** Within-group and between-group differences. BS: Borg scale; 6MWT: six-minute walking test; 30STST: 30-s sit to stand test; Within-group *p*-values come from paired t-test/Z Wilcoxon, based on Shapiro–Wilk *p*-values; between-group *p*-values come from independent t-test/U Mann–Whitney, based on Shapiro–Wilk *p*-values.

	*WITHIN-GROUP*		*BETWEEN-GROUP*
	*EXERCISE*	*CONTROL*	
	*p-Value*		
BS	<0.001	0.712	<0.001
6MWT	0.016	0.993	0.026
30STST	0.011	0.026	0.001

**Table 4:** Between-group differences based on gender. BS: Borg scale; 6MWT: six-minute walking test; 30STST: 30-s sit to stand test; *between-group *p*-values come from the independent t-test*.

	GROUP									
	EXERCISE					CONTROL				
	WOMAN		MEN			WOMAN		MEN		
	M	SD	M	SD	*p*-Value	M	SD	M	SD	*p*-Value
DIF1_BS	−2.56	1.24	−1.89	1.36	0.293	0.20	1.03	−0.12	1.55	0.602
DIF1_6MWT	77.44	105.36	82.11	151.25	0.940	−8.70	16.63	10.75	33.08	0.123
DIF1_30STST	2.44	1.33	0.56	2.55	0.067	−0.60	0.70	−0.50	1.20	0.827

## Data Availability

The study protocol and de-identified individual participant data generated during this study are available from the investigators on reasonable request with the publication. Requests should be directed to the corresponding author by email.

## Supplementary Materials

The following are available online at https://www.mdpi.com/article/10.3390/medicina57070684/s1, CONSORT 2010 checklist of information to include when reporting a pilot or feasibility trial.
